# Paxlovid administration in elderly patient with COVID-19 caused by Omicron BA.2.0: A case report

**DOI:** 10.1097/MD.0000000000031361

**Published:** 2022-11-11

**Authors:** Liulu Zhang, Shasha Zhang, Jing Han, Yile Yi, Hourong Zhou, Jianquan Li

**Affiliations:** a Infectious Departments, Guizhou Provincial People’s Hospital, Guizhou, China; b Department of Radiology, Guizhou Provincial People’s Hospital, Guizhou, China; c Department of Pulmonary and Critical Care Medicine, Guizhou, China; d Department of Cardiovascular Surgery, Guizhou Provincial People’s Hospital, Guizhou, China; e General medicine, Guizhou Provincial People’s Hospital, Guizhou, China; f Intensive Care Unit, Guizhou Provincial People’s Hospital, Guizhou, China.

**Keywords:** COVID-19, elderly patient, Omicron BA 2.0, Paxlovid

## Abstract

Paxlovid has shown the potential decreasing the hospitalization rate of mild or moderate coronavirus disease 2019 (COVID-19) and death in few of clinical trials, and is expected to the most promising medicine targeting Severe Acute Respiratory Syndrome Coronavirus 2 (SRAS-COV-2). However, there are no enough evidences to show it effectiveness for all patients with SARS-COV-2, especially among elderly patients and newest Omicron variant.

**Patient concerns and diagnosis:**

A 79 year’s old female patient was admitted to hospital because of the moderate COVID-19 caused by the Omicron variant BA2.0. He presented the initial syndromes including Xerostomia, cough and fever. Chest computed tomography (CT) scanning at admission showed the exudation lesions on lung. The laboratory examination revealed that there are increased C-reactive protein (CRP), Ferritin and erythrocytesedimentationrate (ESR) and decreased white blood cells.

**Interventions:**

The oral Paxlovid (Nirmatrelvir/Ritonavir) was administrated on second day after admission.

**Outcomes:**

The syndromes of Xerostomia, cough and fever was improved on third day after use of Paxlovid. The levels of CRP, ESR and counts of white blood cells returned the normal after three days of admission. The chest CT scanned on the third and sixth day after Paxlovid used showed the absorption of lesions. The examination of SARS-COVS viral nucleic acid turned negative at fifth day of admission.

**Lessons:**

As a result, we would consider that Paxlovid is a suitable oral drug for elderly patients with SARS-COV2 even Omicron variant, it’s benefit to improve patient’s symptom and signs and can prevents COVID-19 with the high-risk factors from severe disease, although it didn’t shorten the time for viral nucleic acid to turn negative.

## 1. Introduction

Coronavirus disease 2019 (COVID-19) causes the global concerns because of its outbreak spread, till now; there are over 500 million confirmed cases and 6 million death cases among 150 countries and regions around the world. The unpredictable variant of SARS-COV2 remind us that the epidemic is far from ended, and COVID-19 remains a threat in 2022. Based the so high transmission of Severe Acute Respiratory Syndrome Coronavirus 2 (SRAS-COV2) and global health security argument, no-one is safe until everyone is safe.^[1]^

So far, the COVID-19 epidemic has gone through five major SARS-COV2 variants including Alpha, Beta, Gamma, Delta and Omicron. Omicron variant is a newest heavily mutated SARS‐CoV‐2 variant known as B.1.1.529. Existing data suggested that COVID-19 caused by Omicron showing the higher transmission capacity than other SRAS-COV2 variants,^[2–4]^ also WHO announced that the current wave of epidemic mainly resulted from SARS-COV2 Omicron infection. Although early investigation showed that omicron infection results in more asymptomatic or mild COVID-19 cases and links the lower hospitalization rate and death,^[2]^ these reports mainly based the fact that those infected are mainly young population. Thus, this conclusion may mislead, as specialist, Müge Çevik at the University of St Andrews, UK cautioned, these fragmentary of data are not enough to make us lightly treat the impact of such viral infections.^[4]^ More data especially among patients with underlying high risk-factors such as elderly, history disease should be provided around the world.

To date, the exactly mechanism of SARS-COV2 targeting the host cells remains unclear. Currently, most researches tend to consider that the attachment between S protein of SARS-COV2 and angiotensin-converting enzyme-2 (ACE2) receptor of host cells is under mediation of the host protease trans-membrane serine protease2 (TMPRSS2).^[5]^ This procures is followed by the cell fusion and virus replication in host cells, while some protein protease such as papain-likeprotease (Ppro) and Mpro, 3C-like cysteine protease (3CLPro) play the important role in SARS-COV2 viral transcription and replication.^[6,7]^ Thus, any designs acting on these links is likely to achieve the goal of treating the COVID-19. However, several once-promising drugs such as Chloroquine, Remdesivir and Molnupiravir have been proved to be ineffective or limited clinical prospect.^[8]^ Paxlovid is an oral Compound preparation of Nirmatrelvir and Ritonavir targeting SARS-CoV-2 Mpro to inhibit the main protease and present the activity of anti-virus.^[1]^ In several clinic trails, Paxlovid showed the most promise to mild and moderate COVID-19, also is expected to prevent the spread of COVID-19.^[1,6]^ But the effect and safety of Paxlovid against Omicron variant has not yet been reported,^[6]^ especially among populations with high-risk factors such elder, diabetes and other medical history could lead COVID-19 patients entering critical illness.

## 2. Case presentation

**Figure 1.**
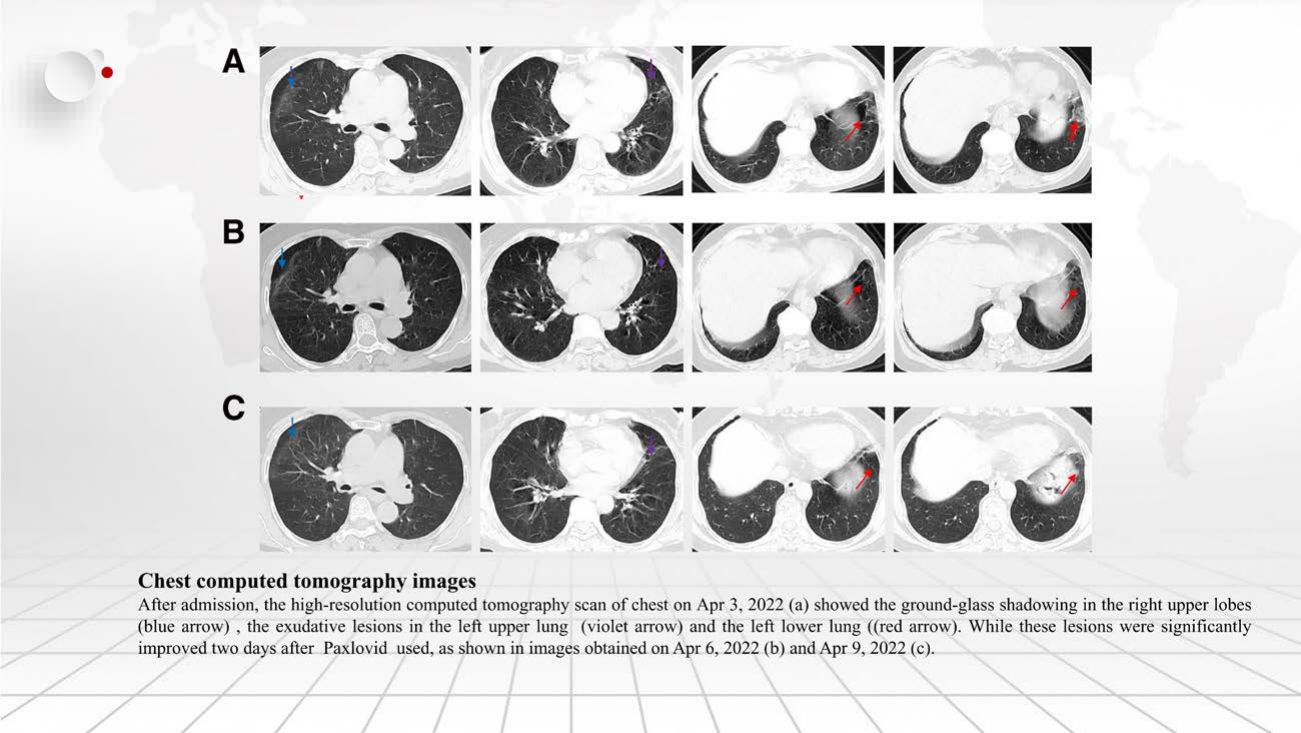
Chest computed tomography images. After admission, the high-resolution computed tomography scan of chest on April 3, 2022 (a) showed the ground-glass shadowing in the right upper lobes (blue arrow), the exudative lesions in the left upper lung (violet arrow) and the left lower lung (red arrow). While these lesions were significantly improved 2 days after Paxlovid used, as shown in images obtained on April 6, 2022 (b) and April 9, 2022 (c).

A 79 years old female patient was admitted to hospital on April 3, 2022 after the confirmed COVID-19 with the positive nucleic acid of SARS-COV2. The sequence analysis of SARS-COV2 showed the Omicron BA 2.0. There was three days of incubation periods before case confirmed. The patient had a history of hypertension for more than 10 years, she denied other medical histories (Table 1) and was not vaccinated against the SARS-COV2. At admission, she presented the symptoms including Xerostomia and cough. Physical examination on admission showed that there were no abnormal signs besides the increased temperature (37.8℃). The examinations of inflammatory parameters revealed that there are increased C-reactive protein (CRP), Ferritin and erythrocytesedimentationrate (ESR) and decreased white blood cells, while other laboratory examinations including hematology, lactate dehydrogenase (LDH), interleukin-6 (IL-6), procalcitonin (PCT) and functions of liver, renal and coagulation were normal (Tables 2-5). The chest computed tomography (CT) scanned on admission showed there are few of infection lesions at the right and left lung (Fig. 1). The main diagnose after admission included: COVID-19 (moderate) and hypertension. The treatments after admission included antiviral therapy with Parovade (Nirmatrelvir 300 mg/Ritonavir 100 mg, Q12 hours for 5 days), prophylactic anticoagulant with low molecular weight heparin calcium (4000 IU, Qd for 5 days), Irbesartan tablets to control blood pressure (75 mg, Qd during hospitalization), and acetylcysteine granules to relieve cough (0.2 g, Tid for 3 days). On third day, the patient’s symptoms of Xerostomia and cough were relieved, chest CT showed that the exudation lesions in lung was significantly absorbed than the previous one. Inflammatory parameters including CRP and ESR returned to almost normal levels after three days. Blood routine examination tested on sixth day showed white blood cells returned to normal (Table 3). While continuous twice (interval 24 hours) of negative viral nucleic acid of SARS-COV2 from nasopharyngeal swab were obtained on the fifth and sixth day (Table 6). Patient discharged on April 20, 2022, during the hospitalization, patient’s liver, renal and coagulation functions were normal.

**Table 1:** Patient’s basic information.

Gender	Female
Age	79 years old
Medical histories	Hypertension for more than 10 years
Incubation period	3 days
Initial symptom	Xerostomia and cough
Initial Ct value of SARS-COVS	
ORF1-a/b	16.75
N	16.96
Vaccination of SARS-COVS	Without vaccination of SRAS-COV2

Ct = Cycle threshold of SARS-COV2.

**Table 2:** The changes of inflammatory parameters.

Time	CRP (mg/L)	IL-6 (pg/mL)	SF (ng/mL)	LDH (U/L)	PCT (ng/mL)	ESR (mm/h)
Day 1	14.15	1.19	445.6	238	0.091	50
Day 3	1.85	3.6	403.1	199	0.056	32
Day 6	2.90	3.626	376.1	174	0.020	63
Reference	0‐8	0‐7	12‐145	115‐220	<0.5	0‐20

CRP = C-reactive protein, ESR = erythrocyte sedimentation rate, IL-6 = interleukin-6, LDH = lactate dehydrogenase, PCT = procalcitonin, SF = serum ferritin.

**Table 3:** The changes of hematology examination.

Time	WBC (×10^9^/L)	LYM (×10^9^/L)	NEUT (×10^9^/L)	NEUT (%)	HB (g/L)	PLT (×10^9^/L)
Day 1	5.09	1.32L	3.22	63.30	162	235
Day 3	3.32	1.38	1.43	43.00	139	224
Day 6	6.61	2.84	3.03	45.80	142	229
Reference	4‐10	0.8‐4	2‐7.7	45‐77	113‐151	100‐300

HB = hemoglobin, LYM = lymphocytotoxicity, NEUT = neutrophile granulocyte, PLT = platelets, WBC = white blood cell.

**Table 4:** The changes of liver and renal function.

Time	CRE (μmol/L)	BUN (mmol/L)	DBILI (μmol/L)	IBILI (μmol/L)	AST (U/L)	ALT (U/L)
Day 1	55.3	5.54	1.8	7.4	43	24
Day 3	45.2	4.36	2.2	8.8	50	33
Day 6	45.2	3.78	1.8	8.6	26	20
Reference	40‐88	2.9‐8.2	0‐6.8	2‐17	8‐40	5‐40

ALT = alanine transaminase, AST = aspartate aminotransferase, BUN = blood urea nitrogen, CRE = Creatinine, DBIL = direct bilirubin, IBILI = indirect bilirubin.

**Table 5:** The changes of blood coagulation function.

Time	PT (s)	APTT (s)	FBG (g/L)	D-D (g/L)
Day 1	12.5	31.2	3.74	0.52
Day 3	11.5	32.0	3.86	0.17
Day 6	11.1	28.2	4.30	0.33
Reference	11‐14.5	26‐44	2‐4	0‐1

APTT = activated partial thromboplastin time, D-D = D-Dimer, FBG = fibrinogen, PT = prothrombin time.

**Table 6:** The Ct of nucleic acid of SARS-COV2.

Time	ORF1ab	N
Day 1	16.75	16.96
Day 3	21.50	25.11
Day 6	29.262	29.262
Day 9	–	35.948
Day 12	–	35.806
Day 13	–	–
Day 14	29.953	35.272
Day 15	–	–
Day 16	–	–
Reference	Negative > 40	Negative > 40

## 3. Discussion

The SARS-CoV-2 has lasted more than two years of global pandemic, and it seriously affects people’s life security and economic and social development. SARS-CoV-2 is genetically variable and so far has experienced 5 main variants of virus: Alpha, Beta, Gamma, Delta and Omicron. The variants of concern (VOCS) such as Delta and Omicron presented the greater threat to health with high infectivity, transmissibility and increased virulence pattern.^[2]^ Although several studies pointed out COVID-19 caused by Omicron showing more asymptomatic or mild cases, it still a threat to public health with its high incidence of transmission.^[4,9,10]^ The mechanism of virus entering and damaging host is intricate and so far not fully understood. It is generally considered that SRAS-COV2 can enter the cells of host by both the mode of endocytosis and membrane fusion after the binding of S proteins of virus with ACE2 receptor of host cells.^[11]^ And then lots of new virus particles formed after experiencing a complex and delicate process including the replication of SARS-COV2 RNA and assembling in rough endoplasmic reticulum (RER) and endoplasmic reticulum golgi intermediate (ERGIC).^[12]^ Followed the replication of SARS-CoV-2 and release via exocytosis, the SARS-CoV-2 damages the host cell through inducing the activation of macrophages and dendritic cells and antigen presentation response of host.^[13–15]^ Due to ACE2 receptors present in various human tissues or organ such as oral cavity, lung, renal, intestinal tract, SARS-COV2 can infect human via various pathways and lead almost all kinds of host cells to damage. However, the latest investigations suggested the mainly injury occurred in respiratory tract, especially the infection caused by Omicron variant.^[13]^ The severe injury causes the alveolar damage and collapse and influences gas exchange, which develops severe disease even death.^[13]^ Not all patients infected with SRAS-COV2 develop the severe disease, some risk-factors and underlying medical histories are associated with severe disease. Lots of researches suggested that older age is one of the identified risk factors associated with poor prognosis and death,^[13,16]^ while the increased levels of aging marker p16, p21 and p53, the higher proportion of aged cells, the decreased of Forkhead box O3 (FOXO3A) in lung tissue and weaker immune-regulatory capacity after SARS-COV2 among older people could be responsible for the severe disease.^[13,16]^ Other medical histories such as hypertension, obesity and diabetes are also considered to be associated with severe disease.^[16]^ Thus, how to prevent COVID-19 patients with these high-risk factors from severe disease is crucial for reducing the mortality. Researches revealed that although the vaccines can’t prevent the infection of SRAS-COV2, it still is the most reliable way to avoid and manage infectious diseases; it can significantly prevent mild case from severe disease.^[9]^ There are no any methods or drugs are thought to completely prevent COVID-19 from developing severe disease or possess the specificity to treat severe disease, especially among those patients with high-risk factors. Several once-promising drugs such as Chloroquine, Remdesivir and Molnupiravir have the limited clinical prospect due to ineffective or side-effect.^[17]^ Paxlovid is a new oral compound of Nirmatrelvir and Ritonavir targeting SARS-CoV-2 Mpro to inhibit the main protease. Among several clinic trails, Paxlovid showed the most promise activity of anti-virus to mild COVID-19, also is expected to prevent the spread of COVID-19. But so far there are no any data showing the potential of Paxlovid against COVID-19 among populations with older age, hypertension and other medical history.

This case study reported an elder COVID-19 patient infected with Omicron BA 2.0. She had the multi-high risk factors developing severe disease including aging, hypertension and without the vaccines of SRAS-COV2. The chest CT scanned on admission has shown the lesions. The use of Paxlovid improved patient’s symptoms and lung lesions and prevent her from severe disease, which could be associated with the effect of Paxlovid blocking virus replication by inhibiting the activation of SARS-CoV-2 Mpro.^[6,7]^ During the hospitalization, we did not find any side-effect of Paxlovid, such as Nausea, vomiting and damage of liver, renal and coagulation function. This is the first report about Paxlovid used in aged patient with SARS-COV2, which provided the reference for treatments among aged people, although there are need more cases and serial of comparative study.

## Author contributions

Liulu Zhang and Shasha Zhang completed the collection of clinical data. Jing Han and Yile Yi contributed to the compilation of data and production of charts. Hourong Zhou and Jianquan Li analyzed all data and wrote the manuscript.

**Data curation:** Jianquan Li, Jing Han, Liu Lu Zhang.

**Formal analysis:** Jianquan Li, Jing Han.

**Investigation:** Jianquan Li, Liu Lu Zhang, Yile Yi.

**Methodology:** Yile Yi.

**Resources:** Hourong Zhou.

**Software:** Shasha Zhang.

**Project administration:** Hourong Zhou, Liu Lu Zhang.

**Visualization:** Shasha Zhang.

**Writing – original draft:** Jianquan Li.

**Writing – review & editing:** Hourong Zhou.
